# Trends in the Epidemiology of Non-Typhoidal Salmonellosis in Israel between 2010 and 2021

**DOI:** 10.3390/ijerph20095626

**Published:** 2023-04-24

**Authors:** Ravit Bassal, Maya Davidovich-Cohen, Eugenia Yakunin, Assaf Rokney, Shifra Ken-Dror, Merav Strauss, Tamar Wolf, Orli Sagi, Sharon Amit, Jacob Moran-Gilad, Orit Treygerman, Racheli Karyo, Lital Keinan-Boker, Dani Cohen

**Affiliations:** 1 Israel Center for Disease Control, Ministry of Health, Sheba Medical Center, Ramat Gan 52621, Israel; 2 Department of Epidemiology and Preventive Medicine, School of Public Health, Sackler Faculty of Medicine, Tel Aviv University, Tel Aviv 69978, Israel; 3 *Salmonella* National Reference Center, Public Health Laboratories-Jerusalem (PHL-J) Public Health Services, Ministry of Health, Jerusalmem 34410, Israel; 4 Microbiology Laboratory, Haifa and Western Gallilee, Clalit Health Services, Nesher 36888, Israel; 5 Microbiology Laboratory, Emek Medical Center, Afula 18341, Israel; 6 Central Laboratory, Maccabi Health Services, Rehovot 76703, Israel; 7 Clinical Microbiology Laboratory, Soroka University Medical Center, Beer-Sheva 84105, Israel; 8 The Faculty of Health Sciences, Ben-Gurion University of the Negev, Beer-Sheva 84105, Israel; 9 Microbiology Laboratories, Sheba Medical Center, Ramat Gan 52621, Israel; 10 Clinical Microbiology Laboratory, The Department of Clinical Microbiology and Infectious Diseases, Hadassah University Hospital, Jerusalem 91120, Israel; 11 Central Laboratory, Meuhedet Health Services, Lod 71293, Israel; 12 Central Laboratory, Clalit Health Services, Tel Aviv 61581, Israel; 13 School of Public Health, University of Haifa, Haifa 34988, Israel

**Keywords:** salmonellosis, epidemiology, incidence rate, Israel

## Abstract

Non-typhoidal salmonellosis (NTS) is one of the most common foodborne diseases worldwide. In this study, we aimed to analyze trends in the epidemiology of NTS in the last decade in Israel. Laboratory-confirmed cases of NTS at eight sentinel laboratories were reported to the Israel Sentinel Laboratory-Based Surveillance Network, integrated with the serotype identification performed at the *Salmonella* National Reference Laboratory of the Ministry of Health. The decrease in NTS incidence since 1999 continued between 2010 and 2014 (16.1 per 100,000 in 2014) and was interrupted by a rise between 2015 and 2017 (39.1 per 100,000 in 2017) associated with outbreaks of *Salmonella* Enteritidis. The incidence of NTS dropped again thereafter (21.4 per 100,000 in 2021). The 0–4 age group was the most affected by NTS (55.5% of the cases) throughout the surveillance period. The age-adjusted incidence rates were consistently high in the summer months (June-September) and low in the winter months (December–February). The overall decrease in the incidence of NTS in Israel since 1999 was temporarily interrupted in the last decade by country-wide outbreaks involving emerging or re-emerging *Salmonella* serotypes. Control measures should be enhanced for all risk points of food chain transmission of *Salmonella* spp. to further reduce the NTS morbidity in Israel.

## 1. Introduction

*Salmonella* is responsible for 9% of the diarrheal illnesses that occur globally each year [1]. *Salmonella* consists of two species, *Salmonella* bongori (*S*. bongori) and *Salmonella* enterica (*S*. enterica), and the latter is further divided into six subspecies that include over 2600 serotypes, based on agglutinating properties of the flagellar H, somatic O, and capsular Vi antigens [2]. Non-typhoidal *Salmonella* (NTS) resides in the intestines of animals and is transmitted to people via the fecal-oral route. Infection may occur directly through the consumption of contaminated food of animal origin (mainly eggs, meat, poultry, and milk), drinking contaminated water, or being in contact with an infected animal or its feces [3].

Human infection by NTS usually manifests as the acute onset of abdominal pain, diarrhea, fever, nausea, and vomiting [3]. The symptoms are usually mild and the disease is self-limiting in most cases, but in some, particularly in children and elderly patients, salmonellosis can be severe and life threatening [3,4]. High-risk populations include children younger than five years of age, the elderly, and immunocompromised individuals [5]. *Salmonella* spp. clinical isolates are most commonly grown from stool specimens but may also be recovered from urine, blood, bones, joints, or the central nervous system.

In Israel, NTS has been a notifiable disease by law since the 1950s and cases of salmonellosis without specification of the *Salmonella* serogroup or serotype are reported passively to the Ministry of Health by physicians and laboratories. To enhance the surveillance of enteric bacterial diseases, including NTS, the Israel Sentinel Laboratory-Based Surveillance Network (ISLBSN) was established in 1997 by the Israel Center for Disease Control (ICDC). The ISLBSN integrates information on *Salmonella* isolates from community and hospital laboratories and from the *Salmonella* National Reference Laboratory, complementing the passive surveillance of salmonellosis. The ISLBSN monitors temporal trends in the incidence of NTS at country or regional level and identifies host- and *Salmonella*-serotype-related risk factors of excess morbidity, counting individual laboratory-proven cases of disease. There is no separate documentation on the number of cases associated with specific outbreaks or clusters of disease.

The generated data help assess and guide existing prevention and control measures of zoonotic transmission of NTS, aiming to reduce the overall burden of the disease in Israel. These include routine testing for NTS, environmental interventions and immunization programs in poultry, and the further prevention of the foodborne transmission of *Salmonella* in humans.

The Israeli Veterinary Services established the Active Surveillance Program to reduce *Salmonella* infection in accordance with the requirements of the European legislation by systematically testing for the presence of *Salmonella* in breeding flocks and in laying hen farms [6]. Chickens identified as carriers of *S*. Typhimurium or *S*. Enteritidis are euthanized. Food products, including dairy, raw vegetables, and fruits from markets, are tested randomly by the National Food Services for contamination with *Salmonella*. In spite of the current preventive measures, NTS is still transmitted to humans when the food chain becomes contaminated, and disease control remains a continuous challenge.

We previously reported on the epidemiology of NTS in Israel between 1999 and 2009 [7]. In this paper, we analyze subsequent trends and epidemiological characteristics of NTS between 2010 and 2021, using the same national sentinel-laboratory-based surveillance network.

## 2. Materials and Methods

Data source: The ISLBSN integrate data on *Salmonella* isolates at sentinel laboratories in Israel and patient demographics with serotype identification performed at the *Salmonella* National Reference Laboratory of the Ministry of Health.

Laboratories included: Five community and three hospital-based laboratories representing the different geographical regions of Israel participate in the ISLBSN (from northern Israel: Haemek Medical Center and Clalit Haifa Health Maintenance Organization (HMO); from central Israel: Sheba Medical Center, Maccabi Dan District HMO, and Clalit Petah-Tikva HMO; from Jerusalem: Hadassah Medical Centers and Meuhedet HMO; from southern Israel: Soroka University Medical Center).

Case definition: A case of NTS was defined as a report on isolation of non-typhoidal *Salmonella* from clinical stool specimens between January 2010 and December 2021 in one of the eight laboratories included in the ISLBSN, located throughout Israel. *Salmonella* samples of the same serogroup and serotype isolated from the same individual within a month were counted only once.

Laboratory methods: Culture-based methods were used by the ISLBSN laboratories for the isolation of *Salmonella* between January 2010 and November 2019. In December 2019, the polymerase chain reaction (PCR) method was introduced gradually for direct detection of *Salmonella* spp. in stool specimens. PCR is used in some of the laboratories as the primary diagnostic tool, followed by culture isolation from PCR-positive samples. Since August 2019, the serotype definition of NTS isolates received at the National Reference Laboratory from sentinel laboratories has comprised two complementary methods: an in-house real-time PCR and the White–Kauffmann–Le Minor serotyping scheme [8]. The laboratories included in the ISLBSN served 50.1% in 2010 and 50.8% in 2021 of the total Israeli population.

Data collection: The data collected for each case included age, gender, birth country, population group, district of residence, and socioeconomic rank information. The socioeconomic rank was defined using the socioeconomic residential classification published in 2015 by the Israeli Central Bureau of Statistic (ICBS), based on 14 variables, including demographic characteristics, education, and lifestyle, with scores ranging between 1, the lowest, and 10, the highest [9]. Based on the ICBS data, the target population served by the ISLBSN was divided by age group (0–4, 5–9, 10–14, 15–19, 20–24, 25–34, 35–44, 45–54, 55–64, and 65+ years), gender (male vs. female), birth country (Israel vs. other), population group (Jews and others: Jews, non-Arabic Christians, and the population with no definition of religion; Arabs: Moslems, Christians, and Druze) and district of residence (Jerusalem, North, Haifa, Central, Tel-Aviv, South and Judea, and Samaria).

Statistical analysis: NTS incidence rates were calculated by dividing the number of non-typhoid *Salmonella* isolates within a specific population group (n) by the total specific population group (N). For the annual age-adjusted incidence rate per 100,000, calculated for 1999–2021, the 2008 Israeli population was used for adjustment, since it represents the mid-point of the surveillance period. For the monthly age-adjusted incidence rate per 100,000 calculated for 2010–2021, the 2015 Israeli population was used for adjustment, since it represents the mid-point of our follow-up. A data analysis was carried out using SAS Enterprise Guide (version 7.12 (7.100.2.3350), SAS Institute Inc., Cary, NC, USA). Secular trends in age-adjusted incidence rates were evaluated between 1999 and 2021 for NTS, using Joinpoint Regression Program (Version 4.9.0.0, Statistical Research and Applications Branch, National Cancer Institute). Slopes between the best-fitting points (joinpoints) of the modeled age-adjusted incidence rates were described by an annual percent change (APC). Significance was defined as *p*-value < 0.05.

## 3. Results

Between January 2010 and December 2021, 11,645 cases of NTS were reported to the ISLBSN. Table 1 presents the characteristics of the cases reported. Most of the cases were reported from Meuhedet HMO (24.3%), from Clalit Haifa HMO (23.4%), and Soroka Medical Center (20.0%) (Table 1). Of the cases reported, 55.5% were infants aged 0–4 and 7.9% were people aged 65+ years, 52.1% were males, 88.8% were born in Israel, 83.2% were Jews and others, 22.8% were residents of Jerusalem district, and 20.7% were residents of the South district. The mean socioeconomic rank was 4.6 (± 2.3 standard deviation).

**Figure 1:**
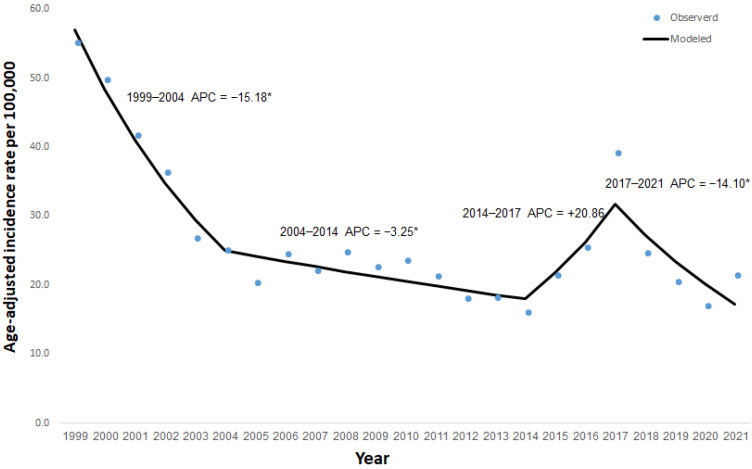
Observed and modeled age-adjusted incidence rates of salmonellosis per 100,000 in Israel, 1999–2021. * *p*-value *<* 0.05.

Figure 1 displays the annual observed age-adjusted incidence rate and the calculated modeled age-adjusted incidence rates of salmonellosis between 1999 and 2021. In 1999, the age-adjusted incidence rate of salmonellosis was 55.2 per 100,000, which decreased to 23.6 per 100,000 in 2010 by the beginning of the surveillance period described in the present study. The decline in the incidence of salmonellosis continued to 16.1 per 100,000 in 2014. This trend was interrupted by the rise in incidence in 2015 and 2016, peaking at 39.1 per 100,000 in 2017. From 2017 to 2021, the incidence of NTS decreased to 21.4 per 100,000 in 2021, a similar incidence rate as in 2010. A relatively low incidence (17.1 per 100,000) was registered in 2020, the first year of the COVID-19 pandemic. Using Joinpoint, we identified four trend periods in the modeled age-adjusted incidence rates: a significant decrease between 1999 and 2004 (APC = −15.18), a significant but moderate decrease between 2004 and 2014 (APC = −3.25), a non-significant increase between 2014 and 2017 (APC = +20.86), and a significant decrease between 2017 and 2021 (APC = −14.10).

**Figure 2:**
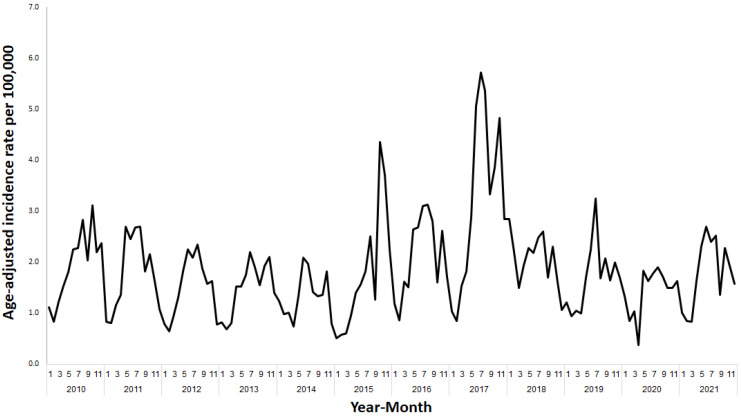
Age-adjusted incidence rate of salmonellosis per 100,000 in Israel by month, January 2010–December 2021.

The age-adjusted incidence rates between January 2010 and December 2021 were high in the summer months (June–September) and lower in the winter months (December–February), demonstrating clear seasonality (Figure 2). Peaks of NTS were observed in October 2015 (4.4 per 100,000) and July 2017 (5.7 per 100,000). The lowest age-adjusted incidence rate was observed in April 2020 (0.4 per 100,000) (Figure 2).

**Figure 3:**
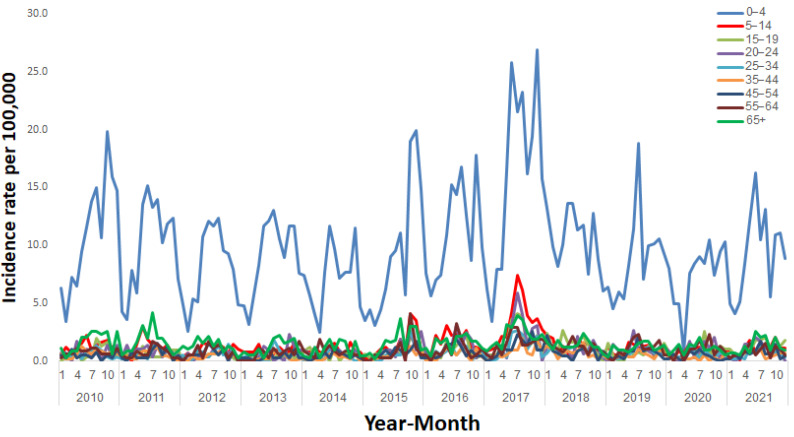
Incidence rates of salmonellosis per 100,000 by age group in Israel, January 2010–December 2021.

The 0–4 age group had the highest NTS age-specific incidence rate, followed by the 65+ years age group (Figure 3).

**Figure 4:**
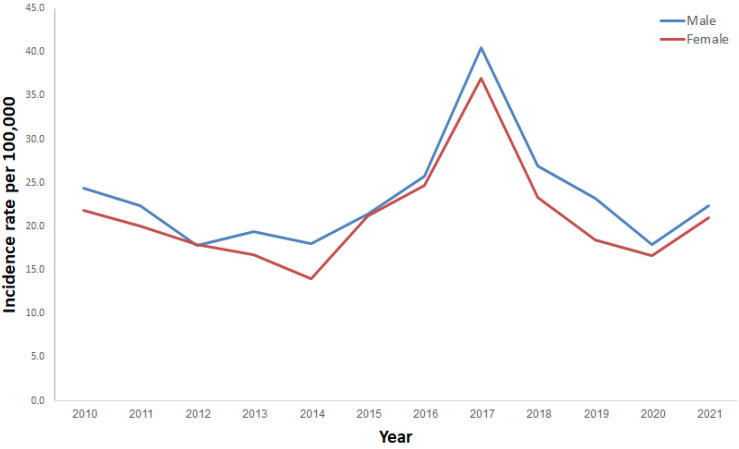
Incidence rates of salmonellosis per 100,000 by gender in Israel, 2010–2021.

A higher NTS rate was observed among males (52.1%) as compared with females (47.9%) across the 2010–2021 surveillance period (*p*-value < 0.0001). This was the result of an excess of cases of NTS in most of the years of the decade (Figure 4).

**Figure 5:**
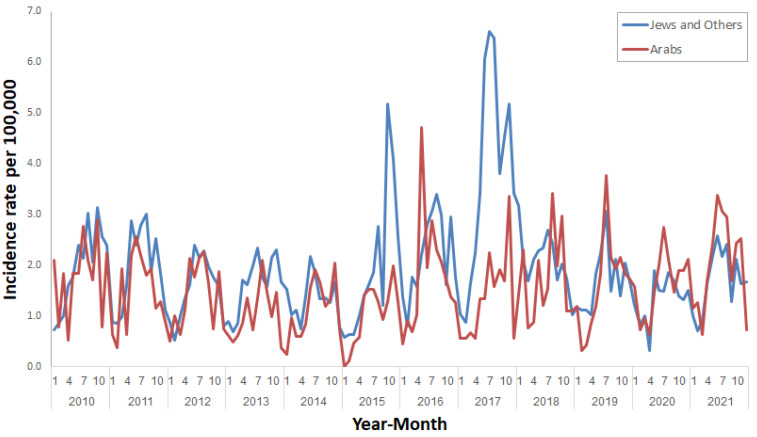
Incidence rates of salmonellosis per 100,000 by population group in Israel, January 2010–December 2021.

Figure 5 illustrates the incidence rates of salmonellosis by population group. Between 2010 and 2014, the incidence rates were almost similar in Jews and others and Arabs. Peaks were observed among Jews and others in October 2015 (5.2 per 100,000), July–August 2017 (6.6 and 6.5 per 100,000, respectively,) and November 2017 (5.2 per 100,000), and among Arabs in May 2016 (4.7 per 100,000). Since 2018, the increase in the incidence rate observed in the summer months has occurred mainly among Arabs.

**Figure 6:**
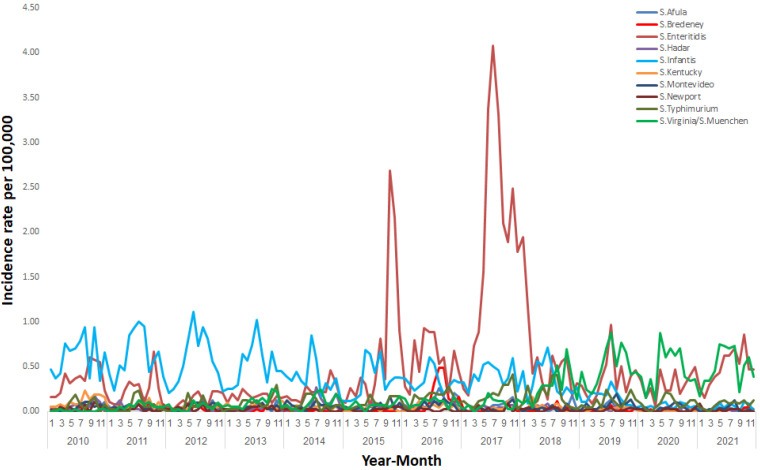
Incidence rates of salmonellosis per 100,000 by serotype in Israel, January 2010–December 2021.

Of the isolates reported to the ISLBSN, 94.4% were submitted to the *Salmonella* Reference Laboratory and were further characterized. The four most prevalent serotypes for the whole surveillance period (2010–2021) were *S*. Enteritidis (28.5%), *S*. Infantis (19.9%), *S*. Virginia or *S*. Muenchen (11.4%), and *S*. Typhimurium (5.9%). Figure 6 shows the trends in the incidence rates of NTS caused by the ten most prevalent serotypes. *S*. Infantis was the dominant serotype causing NTS between 2010 and 2014, with peaks in the summer months. A shift was observed in 2015, when *S*. Enteritidis became the leading serotype. In October 2015 and July 2017, steep increases in the incidence rate of *S*. Enteritidis NTS occurred. Since 2018, the incidence rate of *S*. Virginia or *S*. Muenchen NTS has risen, and in 2020 *S*. Virginia or *S*. Muenchen became the predominant serotype associated with NTS in Israel. Supplementary Materials Table S1 displays the incidence rates of NTS-associated serotypes between 2010 and 2021.

## 4. Discussion

Here, we report on the incidence and epidemiological characteristics of NTS in Israel between 2010 and 2021, based on data generated by the consistent, long-term, national sentinel-laboratory-based surveillance network. The continuous decrease in the incidence of NTS since 1999 was temporarily interrupted in the last decade by two large outbreaks associated with *S*. Enteritidis, the first in 2015, where imported eggs were suspected to be the source of the epidemic agent [10], and the second in 2017 [11], linked to contaminated eggs supplied by a local distributor [12]. A moderate increase in the incidence rate of *S*. Concord in 2018 was associated with Tahini manufactured in Israel [13].

Similar trends of NTS of various serotype etiology were reported by other studies [14,15,16,17,18,19]. In Florida, United States, the annual incidence rate of salmonellosis decreased from 2009 to 2016 and increased in 2017 and 2018 [14]. In New Zealand, a decrease in the incidence rate of non-typhoidal *Salmonella* was reported between 2004 and 2015 [15]. In the EU, the incidence of salmonellosis was stable between 2015 and 2019, after a long period of a declining trend [16]. In Poland, stability of salmonellosis was reported between 2007 and 2017 [17]. In contrast, in Australia, an increase in the incidence rate of salmonellosis was observed between 2000 and 2013 [18]. In India, an increase in the incidence rate of salmonellosis was reported between 2009 and 2018 [19].

According to the current data, the age-adjusted incidence rate of NTS in Israel in 2019 was 20.5 per 100,000, similar to the incidence rate of salmonellosis in the same year in the United States reported by FoodNet (17.1 per 100,000) [20] and in the European Union (EU) (20.0 per 100,000) [16]. This is an important finding, suggesting an improved control of NTS in Israel in the last two decades that has led to a decline in the incidence of the disease to figures comparable with those reported by other OECD countries.

The lowest incidence rate of salmonellosis in 2020 we report here may be attributed to the COVID-19 pandemic. We previously reported and inferred that this was due to the control measures applied in Israel during the COVID-19 pandemic (mostly the lockdowns), with less cases of diarrheal diseases referred to clinics, and to the better performance of laboratory tests including stool cultures in verifying the etiology [21]. Similarly, in Australia, the annual salmonellosis notification rate also decreased by 27% in 2020 compared to the previous 5-year median [22].

We showed that the incidence rates were higher in summer months, as was also reported by others [16,17,23,24]. These findings may be explained by the high temperatures, allowing *Salmonella* to thrive, but also by the behavior of the population during vacations, with people tending to eat outside and being exposed to high-risk food products. The association between salmonellosis and air temperature was demonstrated in the Czech Republic, showing that every 1 °C rise in air temperature contributed to a significant 6.2% increase in salmonellosis [25].

Throughout the study period, the highest NTS morbidity rate was observed among the 0–4 years age group, as was reported previously by us and others [7,14,26], and may be explained by low hygiene practices, the attendance of daycare centers, higher exposure to food items possibly contaminated with *Salmonella*, and higher immunological susceptibility to *Salmonellae* in this very young age group. A weaker immune system associated with aging explains the relatively high incidence of NTC in the 65+ age group as compared with the other age groups except children aged 0–4 years. The bimodal pattern of salmonellosis with the highest incidence rates in children under 5 years of age and in the oldest age group (65+) could be also explained by increased underreporting in the middle age groups, who are less likely to see a physician and be referred to perform a stool culture when experiencing NTS.

We have shown slightly higher incidence rates of NTS among males. Previous studies indicated male gender associated risk excess for NTS and other infectious diseases [27,28]. Peer et al. suggested that genetic and hormonal factors and interactions between hormones and gut microbiota could contribute to the sex differences observed in young children [28].

We found differences in the trends of salmonellosis between Jews and Arabs, while peaks were observed in both population groups throughout the study period. Differences between population groups were also reported by FoodNet using data from 2008 to 2011, generally supporting the trend that minority populations suffer from a greater incidence of salmonellosis [29]. As was reported by FoodNet, it is still not clear whether the differences are a result of underlying cultural food handling practices or alternatively due to socioeconomic factors related to general poor sanitation, pest infestation, and challenges to proper refrigeration, both at the level of the consumer and retail access [29].

The four most prevalent NTS serotypes in Israel, according to the ISLBSN, were *S*. Enteritidis, *S*. Infantis, *S*. Virginia or *S*. Muenchen, and *S*. Typhimurium. *S*. Enteritidis and *S*. Typhimurium were also highly common in the United States [20], China [26], Australia [18], and India [19]. Differences observed in the distribution of *Salmonella* serotypes between countries may be associated with differences in consumption habits. Although *Salmonella* outbreaks are commonly published, 60–80% of all cases are classified as sporadic cases [3]. Egg and egg-based products were reported as the most common food vehicles for *Salmonella* outbreaks [30], and especially common vehicles of epidemic transmission of *S*. Enteritidis NTS [31]. Control measures applied in the veterinary services, including the vaccination of breeding flocks against *S*. Enteritidis and *S*. Typhimurium since the mid-1990s and against *S*. Infantis since 2011, led to a decrease in the overall incidence rate of NTS and presumably to the shift observed between the leading serotypes in Israel in the last decades. Most of the breakthrough morbidity due to *S*. Enteritidis in 2015 to 2017 was associated with the large outbreaks mentioned above, and subsequently the incidence of NTS decreased, continuing the trend that started at the beginning of the millennium.

The significant associations observed in temporal trends between poultry and clinical isolates for *S*. Infantis, *S*. Newport, *S*. Muenchen, and *S*. Virchow indicate that poultry continues to be a significant reservoir for human salmonellosis caused by these four serotypes [32]. Since poultry remains one of the major consumed meat types in most countries, including Israel, primary prevention through a “one-health” strategy must be enhanced to limit the zoonotic transmission of NTS. The predominance of *S*. Enteritidis and *S*. Typhimurium in the poultry production chain has been reduced, resulting in the expansion of previously less common serotypes that are frequently resistant to antibiotics [33]. The emergence of the multi-drug resistant *S*. Virginia or *S*. Muenchen strain, harboring the epidemic megaplasmid, pESI, since 2018 in Israel, as described by Cohen et al. [34], should provide high motivation for highly specific tools for the detection of new spreading strains. Using new assays developed for the detection of not only serotypes but also for distinguishing between specific genetic variants may allow the timely and low-cost detection of emerging strains and potential outbreak cases [35].

It is recommended to further enhance the NTS control measures at all stages of the food chain, from the poultry natural reservoir to the processing, manufacturing, and preparation of foods in both commercial establishments and at home [3]. In Greece, a comprehensive *Salmonella* control program was implemented, including enhanced surveillance on the basis of specific sampling protocols according to EU legislation, vaccination against *S*. Enteritidis and *S*. Typhimurium in poultry, the implementation of strict biosecurity measures, and the restriction of movement and destruction or heat treatment of infected birds and eggs, in the event of the detection of targeted *Salmonella* serotypes in a flock of poultry [36]. All these measures led to a decrease in the incidence of *S*. Enteritidis [36].

Specific guidelines regarding food hygiene, including storage at the right temperature, separating raw meat from cooked food to avoid cross-contamination, hand washing after contacting raw meat, and cooking at correct temperatures, should be re-emphasized to the public before the summer months to prevent a consistent increase in NTS cases in these months.

The strength of our study is the systematic ongoing surveillance network, which has been collecting data since 1997 using the same methodology.

Our study has certain potential limitations. (a) The data included in the ISLBSN are collected from eight laboratories, representing 50.2% of the Israeli population, which may not strictly represent the whole Israeli population, although they cover community and hospitalized cases of salmonellosis with wide geographical locations. (b) The cases reported to the ISLBSN are laboratory confirmed cases, likely underscoring the burden of NTS in the population, as previously estimated [37].

## 5. Conclusions

Our study reiterates the trend of a decline in the incidence of NTS in Israel since 1999, with interruptions and excesses of morbidity associated with the emergence or re-emergence of specific NTS serotypes. Control measures at all stages of the food chain should be enhanced to further reduce the NTS morbidity, especially in the summer months.

## Figures and Tables

**Table 1:** Characteristics of patients with salmonellosis identified through the ISLBSN (January 2010–December 2021).

Variable	Category	N	%
**Sentinel laboratory reporting the isolation of *Salmonella* spp.**	Haemek Medical Center	784	6.7
	Clalit Haifa HMO	2727	23.4
	Maccabi Dan District HMO	1288	11.1
	Soroka Medical Center	2323	20.0
	Sheba Medical Center	291	2.5
	Hadassah Medical Centers	540	4.6
	Meuhedet HMO	2824	24.3
	Clalit Petah-Tikva HMO	868	7.5
	Total	11,645	100.0
**Age group (years)**	0–4	6450	55.5
	5–14	1528	13.1
	15–19	441	3.8
	20–24	431	3.7
	25–34	556	4.8
	35–44	402	3.5
	45–54	397	3.4
	55–64	498	4.3
	65+	922	7.9
**Gender**	Male	6036	52.1
	Female	5557	47.9
**Birth Country**	Israel	10,154	88.8
	Other	1279	11.2
**Population group**	Jews and Others	9263	83.2
	Arabs	1867	16.8
**District**	Jerusalem	2621	22.8
	North	2034	17.7
	Haifa	1488	12.9
	Central	998	8.7
	Tel-Aviv	1234	10.7
	South	2380	20.7
	Judea and Samaria	749	6.5
**Socioeconomic rank**	Mean ± standard deviation	10,164	4.6 ± 2.3

## Data Availability

All relevant data are within the manuscript.

## Supplementary Materials

The following supporting information can be downloaded at: https://www.mdpi.com/article/10.3390/ijerph20095626/s1. Table S1: Incidence rate of salmonellosis per 100,000 by serotype in Israel, 2010–2021.
