# Supplementary material for: Trends in the Epidemiology of Non-Typhoidal Salmonellosis in Israel between 2010 and 2021
Source: Int J Environ Res Public Health. 2023 Apr 24;20(9):5626. doi: 10.3390/ijerph20095626 (PMC10178198; doi:10.3390/ijerph20095626)
Supplement: Supplementary file 1 [file ijerph-20-05626-s001.zip › ijerph-2336591-supplementary.pdf]

Table S1. Incidence rate of salmonellosis per 100,000 by serotype in Israel, 2010-2021..

Serotype	2010	2011	2012	2013	2014	2015	2016	2017	2018	2019	2020	2021
**S.Enteritidis**	4.19	2.65	1.55	1.95	2.50	8.25	6.67	21.97	7.32	4.39	3.62	5.80
**S.Infantis**	7.22	7.19	6.67	6.09	4.32	4.21	4.00	4.65	4.22	1.86	0.77	0.56
**S.Virginia/S.Muenchen**	0.10	0.65	0.59	1.28	1.20	0.81	1.20	0.93	3.28	6.01	5.63	5.93
**S.Typhimurium**	0.76	0.70	0.71	1.13	0.94	1.11	1.81	1.99	2.10	1.36	1.11	0.96
**S.Montevideo**	0.51	0.50	0.64	0.46	0.73	0.55	0.68	0.38	0.31	0.90	0.47	0.30
**S.Kentucky**	1.35	0.70	0.49	0.63	0.52	0.32	0.57	0.51	0.58	0.33	0.02	0.09
**S.Afula**	0.56	0.60	0.44	0.34	0.33	0.37	1.22	0.66	0.40	0.50	0.24	0.41
**S.Hadar**	0.64	0.37	0.32	0.36	0.94	0.35	0.81	0.42	0.42	0.28	0.15	0.09
**S.Bredeney**	0.15	0.42	0.17	0.17	0.07	0.02	1.74	0.31	0.38	0.20	0.11	0.11
**S.Newport**	0.41	0.22	0.37	0.36	0.21	0.30	0.16	0.24	0.25	0.20	0.15	0.17
**S.Virchow**	0.86	0.40	0.49	0.26	0.17	0.09	0.14	0.20	0.09	0.02	0.00	0.06
**S.Havana**	0.03	1.87	0.00	0.39	0.07	0.02	0.05	0.04	0.04	0.02	0.02	0.02
**S.9,12:I,v:-**	0.69	0.45	0.39	0.14	0.09	0.09	0.09	0.09	0.13	0.07	0.06	0.04
**S.Agona**	0.28	0.05	0.20	0.07	0.02	0.21	0.38	0.20	0.31	0.24	0.09	0.19
**S.Mbandaka**	0.41	0.20	0.20	0.34	0.12	0.07	0.23	0.22	0.11	0.15	0.04	0.13
**S.Java**	0.18	0.12	0.10	0.12	0.17	0.18	0.18	0.24	0.42	0.13	0.06	0.13
**S.Saint paul**	0.18	0.10	0.02	0.00	0.07	0.09	0.18	0.09	0.18	0.15	0.34	0.34
**S.Anatum**	0.13	0.25	0.20	0.05	0.21	0.21	0.23	0.16	0.07	0.13	0.02	0.09
**S.Stanley**	0.03	0.32	0.15	0.19	0.19	0.05	0.14	0.18	0.11	0.07	0.02	0.00
**S.Concord**	0.05	0.27	0.10	0.07	0.05	0.09	0.09	0.09	0.31	0.15	0.00	0.15
**S.Tenessee**	0.38	0.12	0.15	0.19	0.07	0.02	0.09	0.02	0.02	0.04	0.02	0.26
**S.Abony**	0.18	0.17	0.15	0.14	0.24	0.02	0.18	0.00	0.09	0.07	0.06	0.09
**S.Senftenberg**	0.33	0.05	0.15	0.19	0.17	0.14	0.11	0.11	0.07	0.02	0.00	0.02
**S.16:I,v:-**	0.00	0.02	0.25	0.00	0.05	0.00	0.05	0.09	0.16	0.13	0.13	0.04
**S.II 47:b:e,n,x,z15**	0.13	0.02	0.12	0.05	0.02	0.07	0.23	0.11	0.09	0.04	0.00	0.00
**S.Eastbourne**	0.00	0.02	0.00	0.00	0.21	0.12	0.23	0.07	0.11	0.09	0.02	0.00
**S.13:23:I:-**	0.23	0.15	0.05	0.00	0.00	0.02	0.09	0.00	0.04	0.11	0.09	0.00
**S.2(nachsonim)**	0.10	0.07	0.10	0.10	0.07	0.00	0.11	0.07	0.09	0.02	0.00	0.04
**S.Rissen**	0.05	0.07	0.00	0.05	0.00	0.07	0.02	0.24	0.13	0.13	0.00	0.00
**S.Manhattan**	0.05	0.02	0.02	0.19	0.17	0.07	0.02	0.13	0.02	0.00	0.04	0.02
**S.Cleveland**	0.00	0.00	0.00	0.00	0.00	0.21	0.43	0.09	0.00	0.00	0.00	0.00
**S.Kottbus**	0.05	0.02	0.07	0.14	0.05	0.07	0.05	0.07	0.11	0.00	0.04	0.02
**S.Coeln**	0.00	0.00	0.02	0.00	0.02	0.16	0.09	0.24	0.02	0.04	0.04	0.02
**S.Hvittingfoss**	0.33	0.02	0.00	0.00	0.00	0.05	0.02	0.18	0.04	0.02	0.00	0.00
**S.4,12:i:-**	0.15	0.10	0.05	0.10	0.02	0.02	0.05	0.07	0.07	0.00	0.02	0.02
**S.Richmond**	0.08	0.15	0.02	0.05	0.05	0.02	0.05	0.07	0.02	0.02	0.00	0.13
**S.Blockley**	0.05	0.05	0.15	0.19	0.09	0.05	0.05	0.00	0.02	0.00	0.00	0.00
**S.Rough:r:1,5**	0.08	0.05	0.10	0.07	0.07	0.05	0.11	0.00	0.02	0.04	0.00	0.00
**S.4,5,12:i:-**	0.00	0.00	0.00	0.00	0.00	0.00	0.00	0.02	0.16	0.20	0.06	0.15
**S.Oranienburg**	0.00	0.02	0.07	0.00	0.02	0.05	0.11	0.04	0.02	0.07	0.11	0.04
**S.Bareilly**	0.08	0.02	0.12	0.00	0.00	0.12	0.14	0.02	0.02	0.02	0.00	0.02
**S.Livingstone**	0.00	0.02	0.05	0.00	0.00	0.00	0.23	0.07	0.07	0.02	0.00	0.04
**S.Poona**	0.00	0.05	0.00	0.05	0.02	0.07	0.00	0.07	0.07	0.02	0.02	0.11

**S.Give**	0.00	0.00	0.00	0.00	0.12	0.00	0.07	0.04	0.13	0.07	0.04	0.00
**S.Bovis-morbificans**	0.03	0.00	0.00	0.00	0.00	0.00	0.02	0.11	0.07	0.11	0.09	0.04
**S.II(uphill)**	0.05	0.00	0.02	0.05	0.07	0.07	0.00	0.09	0.09	0.00	0.02	0.00
**S.Braenderup**	0.10	0.00	0.02	0.00	0.02	0.05	0.11	0.04	0.02	0.04	0.00	0.00
**S.Corvallis**	0.10	0.10	0.07	0.02	0.00	0.02	0.00	0.07	0.02	0.00	0.00	0.00
**S.Muenster**	0.08	0.00	0.02	0.10	0.00	0.00	0.07	0.04	0.07	0.02	0.00	0.00
**S.II(negev)**	0.00	0.02	0.05	0.05	0.00	0.05	0.07	0.02	0.07	0.07	0.00	0.00
**S.Altona**	0.00	0.00	0.07	0.00	0.02	0.09	0.07	0.07	0.02	0.00	0.00	0.02
**S.Cubana**	0.03	0.02	0.17	0.12	0.02	0.00	0.00	0.00	0.00	0.00	0.00	0.00
**S.Edinburg**	0.10	0.02	0.20	0.00	0.00	0.00	0.00	0.00	0.02	0.00	0.00	0.00
**S.Schwarzengrund**	0.05	0.07	0.07	0.02	0.02	0.02	0.05	0.00	0.00	0.00	0.02	0.00
**S.Tarshyne**	0.08	0.02	0.00	0.10	0.02	0.02	0.00	0.02	0.02	0.00	0.00	0.02
**S.Meleagridis**	0.03	0.12	0.00	0.00	0.00	0.05	0.00	0.00	0.07	0.02	0.00	0.02
**S.Orion**	0.00	0.00	0.02	0.00	0.00	0.00	0.02	0.04	0.00	0.00	0.00	0.21
**S.Reading**	0.03	0.00	0.00	0.02	0.00	0.05	0.05	0.02	0.04	0.04	0.02	0.02
**S.Liverpool**	0.00	0.02	0.00	0.00	0.02	0.02	0.05	0.07	0.00	0.04	0.04	0.02
**S.Kedougou**	0.00	0.00	0.00	0.02	0.00	0.02	0.00	0.02	0.16	0.00	0.04	0.02
**S.Cerro**	0.00	0.07	0.05	0.02	0.02	0.02	0.02	0.04	0.02	0.00	0.00	0.00
**S.Rough**	0.03	0.02	0.05	0.05	0.02	0.00	0.05	0.02	0.00	0.00	0.00	0.00
**S.2[sofia]**	0.05	0.00	0.07	0.00	0.00	0.00	0.00	0.00	0.04	0.07	0.00	0.00
**S.Freetown**	0.03	0.02	0.00	0.02	0.02	0.02	0.00	0.02	0.00	0.00	0.06	0.00
**S.Goeteborg**	0.03	0.05	0.00	0.00	0.00	0.02	0.00	0.04	0.02	0.02	0.00	0.00
**S.Derby**	0.03	0.00	0.00	0.02	0.00	0.05	0.00	0.00	0.02	0.02	0.02	0.02
**S.IIIb 61:i:z53**	0.03	0.00	0.00	0.00	0.00	0.02	0.00	0.00	0.02	0.00	0.00	0.11
**S.Colindale**	0.00	0.00	0.02	0.07	0.05	0.02	0.00	0.00	0.00	0.00	0.00	0.00
**S.Charity**	0.05	0.00	0.00	0.00	0.02	0.05	0.00	0.04	0.00	0.00	0.00	0.00
**S.Idikan**	0.03	0.00	0.05	0.00	0.00	0.02	0.00	0.02	0.02	0.02	0.00	0.00
**S.Amager**	0.03	0.00	0.02	0.00	0.05	0.00	0.00	0.00	0.00	0.00	0.06	0.00
**S.Ohio**	0.00	0.00	0.02	0.00	0.02	0.00	0.02	0.00	0.04	0.02	0.02	0.00
**S.Heidelberg**	0.05	0.02	0.00	0.02	0.00	0.02	0.00	0.00	0.02	0.00	0.00	0.00
**S.Paratyphi B**	0.00	0.00	0.02	0.00	0.05	0.02	0.02	0.00	0.00	0.02	0.00	0.00
**S.Haifa**	0.00	0.02	0.02	0.02	0.00	0.00	0.00	0.00	0.02	0.00	0.04	0.00
**S.Weltevreden**	0.00	0.00	0.00	0.02	0.02	0.02	0.05	0.00	0.00	0.02	0.00	0.00
**S.Isangi**	0.00	0.00	0.02	0.00	0.00	0.00	0.00	0.00	0.00	0.11	0.00	0.00
**S.4,12:-:1,2**	0.00	0.00	0.00	0.00	0.00	0.00	0.05	0.02	0.00	0.00	0.00	0.06
**S.Hindmarsh**	0.00	0.00	0.00	0.00	0.02	0.00	0.00	0.00	0.00	0.04	0.02	0.04
**S.Sandiego**	0.05	0.00	0.00	0.05	0.02	0.00	0.00	0.00	0.00	0.00	0.00	0.00
**S.8,20:-:z6**	0.03	0.00	0.02	0.02	0.02	0.02	0.00	0.00	0.00	0.00	0.00	0.00
**S.Postdam**	0.03	0.00	0.05	0.00	0.00	0.02	0.00	0.00	0.00	0.02	0.00	0.00
**S.Goldcoast**	0.03	0.02	0.00	0.00	0.02	0.00	0.02	0.00	0.00	0.00	0.00	0.02
**S.II(hagenbeck)**	0.00	0.00	0.05	0.00	0.02	0.02	0.00	0.00	0.00	0.02	0.00	0.00
**S.Istanbul**	0.00	0.00	0.00	0.00	0.00	0.05	0.02	0.02	0.02	0.00	0.00	0.00
**S.Worthington**	0.00	0.00	0.00	0.00	0.00	0.00	0.00	0.00	0.11	0.00	0.00	0.00
**S.Ealing**	0.00	0.00	0.00	0.00	0.00	0.00	0.00	0.07	0.04	0.00	0.00	0.00

**S.6,8:r:-**	0.00	0.00	0.00	0.00	0.00	0.00	0.00	0.00	0.00	0.00	0.00	0.11
**S.Brancaster**	0.00	0.00	0.00	0.00	0.00	0.00	0.00	0.00	0.00	0.00	0.00	0.11
**S.Wangata**	0.05	0.00	0.05	0.00	0.00	0.00	0.00	0.00	0.00	0.00	0.00	0.00
**S.Brandenburg**	0.00	0.00	0.02	0.02	0.00	0.02	0.00	0.00	0.00	0.02	0.00	0.00
**S.6,7:f,g,t:-**	0.00	0.00	0.00	0.05	0.00	0.00	0.02	0.00	0.00	0.02	0.00	0.00
**S.Paratyphi A**	0.00	0.00	0.02	0.00	0.00	0.00	0.05	0.02	0.00	0.00	0.00	0.00
**S.Vejle**	0.00	0.00	0.00	0.05	0.00	0.00	0.00	0.00	0.00	0.00	0.02	0.02
**S.Rubislaw**	0.03	0.00	0.00	0.00	0.00	0.00	0.00	0.00	0.02	0.00	0.04	0.00
**S.Thompson**	0.00	0.00	0.00	0.00	0.00	0.00	0.05	0.02	0.00	0.00	0.02	0.00
**S.Uganda**	0.00	0.00	0.00	0.00	0.00	0.00	0.00	0.02	0.02	0.04	0.00	0.00
**S.Bonn**	0.00	0.00	0.00	0.00	0.00	0.00	0.00	0.00	0.04	0.02	0.00	0.02
**S.4,5,12:-:1,2**	0.00	0.00	0.00	0.00	0.00	0.00	0.00	0.00	0.00	0.00	0.09	0.00
**S.8:rough**	0.00	0.00	0.00	0.00	0.00	0.00	0.00	0.00	0.00	0.00	0.00	0.09
**S.6,7:z10:-**	0.00	0.05	0.00	0.00	0.02	0.00	0.00	0.00	0.00	0.00	0.00	0.00
**S.4,12: rough**	0.00	0.00	0.05	0.02	0.00	0.00	0.00	0.00	0.00	0.00	0.00	0.00
**S.6,8:z10:-**	0.03	0.00	0.02	0.00	0.00	0.02	0.00	0.00	0.00	0.00	0.00	0.00
**S.4,12:b:-**	0.03	0.02	0.00	0.00	0.00	0.00	0.02	0.00	0.00	0.00	0.00	0.00
**S.Panama**	0.00	0.00	0.02	0.02	0.00	0.02	0.00	0.00	0.00	0.00	0.00	0.00
**S.Oslo**	0.00	0.00	0.02	0.00	0.00	0.05	0.00	0.00	0.00	0.00	0.00	0.00
**S.Alachua**	0.03	0.00	0.00	0.00	0.00	0.00	0.02	0.00	0.00	0.02	0.00	0.00
**S.Stanleyvill**	0.00	0.00	0.02	0.00	0.00	0.00	0.00	0.02	0.02	0.00	0.00	0.00
**S.Yoruba**	0.00	0.00	0.00	0.00	0.00	0.00	0.07	0.00	0.00	0.00	0.00	0.00
**S.III b 50 l,v e,n,x,z15**	0.00	0.00	0.00	0.00	0.00	0.02	0.02	0.00	0.00	0.00	0.00	0.02
**S.Damman**	0.00	0.00	0.00	0.00	0.00	0.00	0.02	0.02	0.00	0.00	0.00	0.02
**S.9,12:-:1,5**	0.00	0.00	0.00	0.00	0.00	0.00	0.00	0.04	0.00	0.02	0.00	0.00
**S.Kalina**	0.05	0.00	0.00	0.00	0.00	0.00	0.00	0.00	0.00	0.00	0.00	0.00
**S.4,12:-:-**	0.00	0.02	0.02	0.00	0.00	0.00	0.00	0.00	0.00	0.00	0.00	0.00
**S.Emek**	0.00	0.02	0.02	0.00	0.00	0.00	0.00	0.00	0.00	0.00	0.00	0.00
**S.6,7:r:-**	0.00	0.00	0.02	0.02	0.00	0.00	0.00	0.00	0.00	0.00	0.00	0.00
**S.Albany**	0.00	0.02	0.00	0.00	0.02	0.00	0.00	0.00	0.00	0.00	0.00	0.00
**S.Wandsworth**	0.03	0.00	0.00	0.00	0.00	0.02	0.00	0.00	0.00	0.00	0.00	0.00
**S.Wagenia**	0.00	0.00	0.00	0.05	0.00	0.00	0.00	0.00	0.00	0.00	0.00	0.00
**S.16:-:-**	0.00	0.02	0.00	0.00	0.00	0.02	0.00	0.00	0.00	0.00	0.00	0.00
**S.II 42:z10:1,2**	0.00	0.00	0.02	0.00	0.00	0.02	0.00	0.00	0.00	0.00	0.00	0.00
**S.Glostrup**	0.03	0.00	0.00	0.00	0.00	0.00	0.00	0.02	0.00	0.00	0.00	0.00
**S.Telelkebir**	0.00	0.00	0.00	0.02	0.00	0.00	0.02	0.00	0.00	0.00	0.00	0.00
**S.II 40:Z4,Z24:Z39**	0.00	0.00	0.02	0.00	0.00	0.00	0.00	0.00	0.00	0.02	0.00	0.00
**S.Dublin**	0.00	0.02	0.00	0.00	0.00	0.00	0.00	0.00	0.00	0.00	0.02	0.00
**S.Durban**	0.00	0.02	0.00	0.00	0.00	0.00	0.00	0.00	0.00	0.00	0.02	0.00
**S.Molade**	0.00	0.00	0.00	0.00	0.00	0.02	0.02	0.00	0.00	0.00	0.00	0.00
**S.Urbana**	0.00	0.00	0.00	0.02	0.00	0.00	0.00	0.00	0.00	0.00	0.00	0.02
**S.6,7:-:-**	0.00	0.00	0.00	0.00	0.02	0.00	0.00	0.00	0.00	0.02	0.00	0.00
**S.Napoli**	0.00	0.00	0.00	0.00	0.00	0.02	0.00	0.00	0.02	0.00	0.00	0.00
**S.Rough:k:1,5**	0.00	0.00	0.00	0.00	0.00	0.00	0.05	0.00	0.00	0.00	0.00	0.00

**S.Eingedi**	0.00	0.00	0.00	0.00	0.00	0.00	0.02	0.02	0.00	0.00	0.00	0.00
**S.IIIb 35 z52 e,n,x,z15**	0.00	0.00	0.00	0.00	0.00	0.00	0.02	0.02	0.00	0.00	0.00	0.00
**S.Kingabwa**	0.00	0.00	0.00	0.00	0.00	0.00	0.00	0.00	0.04	0.00	0.00	0.00
**S.Bardo**	0.00	0.00	0.00	0.00	0.00	0.02	0.00	0.00	0.00	0.00	0.00	0.02
**S.IIIb 50:k:z35**	0.00	0.00	0.00	0.00	0.00	0.02	0.00	0.00	0.00	0.00	0.00	0.02
**S.8,20:i:-**	0.00	0.00	0.00	0.00	0.00	0.00	0.00	0.02	0.02	0.00	0.00	0.00
**S.Indiana**	0.00	0.00	0.00	0.00	0.00	0.00	0.00	0.02	0.02	0.00	0.00	0.00
**S.IIIb 48 k z53**	0.00	0.00	0.00	0.00	0.00	0.00	0.00	0.04	0.00	0.00	0.00	0.00
**S.Ahuza**	0.00	0.00	0.00	0.00	0.00	0.00	0.02	0.00	0.00	0.00	0.00	0.02
**S.Litchfield**	0.00	0.00	0.00	0.00	0.00	0.00	0.02	0.00	0.00	0.00	0.00	0.02
**S.8:-:-**	0.00	0.00	0.00	0.00	0.00	0.00	0.00	0.02	0.00	0.02	0.00	0.00
**S.Umbilo**	0.00	0.00	0.00	0.00	0.00	0.00	0.00	0.02	0.00	0.02	0.00	0.00
**S.Falkensee**	0.00	0.00	0.00	0.00	0.00	0.00	0.00	0.00	0.02	0.00	0.00	0.02
**S.Rough:d:1,2**	0.00	0.00	0.00	0.00	0.00	0.00	0.00	0.00	0.00	0.02	0.00	0.02
**S.4,12:b:Rough**	0.03	0.00	0.00	0.00	0.00	0.00	0.00	0.00	0.00	0.00	0.00	0.00
**S.Johannesburg**	0.03	0.00	0.00	0.00	0.00	0.00	0.00	0.00	0.00	0.00	0.00	0.00
**S.Malstatt**	0.03	0.00	0.00	0.00	0.00	0.00	0.00	0.00	0.00	0.00	0.00	0.00
**S.Nyanza**	0.03	0.00	0.00	0.00	0.00	0.00	0.00	0.00	0.00	0.00	0.00	0.00
**S.Rough:g,s,t:-**	0.03	0.00	0.00	0.00	0.00	0.00	0.00	0.00	0.00	0.00	0.00	0.00
**S.Vitkin**	0.03	0.00	0.00	0.00	0.00	0.00	0.00	0.00	0.00	0.00	0.00	0.00
**S.4,5,12:-:-**	0.00	0.02	0.00	0.00	0.00	0.00	0.00	0.00	0.00	0.00	0.00	0.00
**S.II 41:Z10:-**	0.00	0.02	0.00	0.00	0.00	0.00	0.00	0.00	0.00	0.00	0.00	0.00
**S.Lexington**	0.00	0.02	0.00	0.00	0.00	0.00	0.00	0.00	0.00	0.00	0.00	0.00
**S.Rough:r:1,2**	0.00	0.02	0.00	0.00	0.00	0.00	0.00	0.00	0.00	0.00	0.00	0.00
**S.Rough:b:1,2**	0.00	0.02	0.00	0.00	0.00	0.00	0.00	0.00	0.00	0.00	0.00	0.00
**S.6,7:b:-**	0.00	0.00	0.02	0.00	0.00	0.00	0.00	0.00	0.00	0.00	0.00	0.00
**S.6:8:-:-**	0.00	0.00	0.02	0.00	0.00	0.00	0.00	0.00	0.00	0.00	0.00	0.00
**S.London**	0.00	0.00	0.02	0.00	0.00	0.00	0.00	0.00	0.00	0.00	0.00	0.00
**S.Rough:1,5**	0.00	0.00	0.02	0.00	0.00	0.00	0.00	0.00	0.00	0.00	0.00	0.00
**S.Toricada**	0.00	0.00	0.02	0.00	0.00	0.00	0.00	0.00	0.00	0.00	0.00	0.00
**S.9,12:z66:-**	0.00	0.00	0.00	0.02	0.00	0.00	0.00	0.00	0.00	0.00	0.00	0.00
**S.Lawra**	0.00	0.00	0.00	0.02	0.00	0.00	0.00	0.00	0.00	0.00	0.00	0.00
**S.Mkamba**	0.00	0.00	0.00	0.02	0.00	0.00	0.00	0.00	0.00	0.00	0.00	0.00
**S.Shubra**	0.00	0.00	0.00	0.02	0.00	0.00	0.00	0.00	0.00	0.00	0.00	0.00
**S.Bere**	0.00	0.00	0.00	0.00	0.02	0.00	0.00	0.00	0.00	0.00	0.00	0.00
**S.Grumpensis**	0.00	0.00	0.00	0.00	0.02	0.00	0.00	0.00	0.00	0.00	0.00	0.00
**S.Halle**	0.00	0.00	0.00	0.00	0.02	0.00	0.00	0.00	0.00	0.00	0.00	0.00
**S.IIIb 38 l,v z35**	0.00	0.00	0.00	0.00	0.02	0.00	0.00	0.00	0.00	0.00	0.00	0.00
**S.Ituri**	0.00	0.00	0.00	0.00	0.02	0.00	0.00	0.00	0.00	0.00	0.00	0.00
**S.Kasenyi**	0.00	0.00	0.00	0.00	0.02	0.00	0.00	0.00	0.00	0.00	0.00	0.00
**S.Kiambu**	0.00	0.00	0.00	0.00	0.02	0.00	0.00	0.00	0.00	0.00	0.00	0.00
**S.Leeuwarden**	0.00	0.00	0.00	0.00	0.02	0.00	0.00	0.00	0.00	0.00	0.00	0.00
**S.3,10:e,h:-**	0.00	0.00	0.00	0.00	0.00	0.02	0.00	0.00	0.00	0.00	0.00	0.00
**S.6,7:k:-**	0.00	0.00	0.00	0.00	0.00	0.02	0.00	0.00	0.00	0.00	0.00	0.00

**S.Ball**	0.00	0.00	0.00	0.00	0.00	0.02	0.00	0.00	0.00	0.00	0.00	0.00
**S.IIIb 48 i z [z72]**	0.00	0.00	0.00	0.00	0.00	0.02	0.00	0.00	0.00	0.00	0.00	0.00
**S.IIIb:63:-:1,5**	0.00	0.00	0.00	0.00	0.00	0.02	0.00	0.00	0.00	0.00	0.00	0.00
**S.Matopenni**	0.00	0.00	0.00	0.00	0.00	0.02	0.00	0.00	0.00	0.00	0.00	0.00
**S.Monschaui**	0.00	0.00	0.00	0.00	0.00	0.02	0.00	0.00	0.00	0.00	0.00	0.00
**S.Tananarive**	0.00	0.00	0.00	0.00	0.00	0.02	0.00	0.00	0.00	0.00	0.00	0.00
**S.Yolo**	0.00	0.00	0.00	0.00	0.00	0.02	0.00	0.00	0.00	0.00	0.00	0.00
**S,6,8:-:1,2**	0.00	0.00	0.00	0.00	0.00	0.00	0.02	0.00	0.00	0.00	0.00	0.00
**S.1,3,19:f,g,t:-**	0.00	0.00	0.00	0.00	0.00	0.00	0.02	0.00	0.00	0.00	0.00	0.00
**S.Chester**	0.00	0.00	0.00	0.00	0.00	0.00	0.02	0.00	0.00	0.00	0.00	0.00
**S.Mgulani**	0.00	0.00	0.00	0.00	0.00	0.00	0.02	0.00	0.00	0.00	0.00	0.00
**S.Quentin**	0.00	0.00	0.00	0.00	0.00	0.00	0.02	0.00	0.00	0.00	0.00	0.00
**S.Welikade**	0.00	0.00	0.00	0.00	0.00	0.00	0.02	0.00	0.00	0.00	0.00	0.00
**S.Widemarsh**	0.00	0.00	0.00	0.00	0.00	0.00	0.02	0.00	0.00	0.00	0.00	0.00
**IIIa 50:k:-**	0.00	0.00	0.00	0.00	0.00	0.00	0.00	0.00	0.02	0.00	0.00	0.00
**S.II 23:z:1,5**	0.00	0.00	0.00	0.00	0.00	0.00	0.00	0.00	0.02	0.00	0.00	0.00
**S.IIIa 48:z4,z23:–**	0.00	0.00	0.00	0.00	0.00	0.00	0.00	0.00	0.02	0.00	0.00	0.00
**S.Javiana**	0.00	0.00	0.00	0.00	0.00	0.00	0.00	0.00	0.02	0.00	0.00	0.00
**S.Mikawasima**	0.00	0.00	0.00	0.00	0.00	0.00	0.00	0.00	0.02	0.00	0.00	0.00
**S.Nima**	0.00	0.00	0.00	0.00	0.00	0.00	0.00	0.00	0.02	0.00	0.00	0.00
**S.13,23:-:-**	0.00	0.00	0.00	0.00	0.00	0.00	0.00	0.02	0.00	0.00	0.00	0.00
**S.6,14,25:-:1,5**	0.00	0.00	0.00	0.00	0.00	0.00	0.00	0.02	0.00	0.00	0.00	0.00
**S.6,7:–:1,5**	0.00	0.00	0.00	0.00	0.00	0.00	0.00	0.02	0.00	0.00	0.00	0.00
**S.Ezra**	0.00	0.00	0.00	0.00	0.00	0.00	0.00	0.02	0.00	0.00	0.00	0.00
**S.Gombe**	0.00	0.00	0.00	0.00	0.00	0.00	0.00	0.02	0.00	0.00	0.00	0.00
**S.II 17 b e,n,x,z15**	0.00	0.00	0.00	0.00	0.00	0.00	0.00	0.02	0.00	0.00	0.00	0.00
**S.Nottingham**	0.00	0.00	0.00	0.00	0.00	0.00	0.00	0.02	0.00	0.00	0.00	0.00
**S.Ouakam**	0.00	0.00	0.00	0.00	0.00	0.00	0.00	0.02	0.00	0.00	0.00	0.00
**S.Rough:g,m:-**	0.00	0.00	0.00	0.00	0.00	0.00	0.00	0.02	0.00	0.00	0.00	0.00
**S.Rough:i,v:1,7**	0.00	0.00	0.00	0.00	0.00	0.00	0.00	0.02	0.00	0.00	0.00	0.00
**S.Westhampton**	0.00	0.00	0.00	0.00	0.00	0.00	0.00	0.02	0.00	0.00	0.00	0.00
**S.Zanzibar**	0.00	0.00	0.00	0.00	0.00	0.00	0.00	0.02	0.00	0.00	0.00	0.00
**S.4,12:y:-**	0.00	0.00	0.00	0.00	0.00	0.00	0.00	0.00	0.00	0.02	0.00	0.00
**S.40:a:-**	0.00	0.00	0.00	0.00	0.00	0.00	0.00	0.00	0.00	0.02	0.00	0.00
**S.9,12:I,z13:-**	0.00	0.00	0.00	0.00	0.00	0.00	0.00	0.00	0.00	0.02	0.00	0.00
**S.Baildon 9, 46 a e, n, x**	0.00	0.00	0.00	0.00	0.00	0.00	0.00	0.00	0.00	0.02	0.00	0.00
**S.IIIb 50:l,z13:e,n,z15**	0.00	0.00	0.00	0.00	0.00	0.00	0.00	0.00	0.00	0.02	0.00	0.00
**S.Veneziana**	0.00	0.00	0.00	0.00	0.00	0.00	0.00	0.00	0.00	0.02	0.00	0.00
**S,9,12:rough**	0.00	0.00	0.00	0.00	0.00	0.00	0.00	0.00	0.00	0.00	0.00	0.02
**S.8,20:rough**	0.00	0.00	0.00	0.00	0.00	0.00	0.00	0.00	0.00	0.00	0.00	0.02
**S.Abortusequi**	0.00	0.00	0.00	0.00	0.00	0.00	0.00	0.00	0.00	0.00	0.00	0.02
**S.Carmel**	0.00	0.00	0.00	0.00	0.00	0.00	0.00	0.00	0.00	0.00	0.02	0.00
**S.Ferruch**	0.00	0.00	0.00	0.00	0.00	0.00	0.00	0.00	0.00	0.00	0.00	0.02
**S.Haardt**	0.00	0.00	0.00	0.00	0.00	0.00	0.00	0.00	0.00	0.00	0.00	0.02

**S.IIIb 13, 23 z 1, 5**	0.00	0.00	0.00	0.00	0.00	0.00	0.00	0.00	0.00	0.00	0.02	0.00
**S.IIIb 38 l, v [z53] [z54]**	0.00	0.00	0.00	0.00	0.00	0.00	0.00	0.00	0.00	0.00	0.02	0.00
**S.IIIb 61:-:1, 5**	0.00	0.00	0.00	0.00	0.00	0.00	0.00	0.00	0.00	0.00	0.00	0.02
**S.IIIb:65:z52 z**	0.00	0.00	0.00	0.00	0.00	0.00	0.00	0.00	0.00	0.00	0.02	0.00
**S.Kaevlinge**	0.00	0.00	0.00	0.00	0.00	0.00	0.00	0.00	0.00	0.00	0.02	0.00
**S.Milwaukee**	0.00	0.00	0.00	0.00	0.00	0.00	0.00	0.00	0.00	0.00	0.02	0.00
**S.Mishmarhaemek**	0.00	0.00	0.00	0.00	0.00	0.00	0.00	0.00	0.00	0.00	0.02	0.00
**S.Pakistan**	0.00	0.00	0.00	0.00	0.00	0.00	0.00	0.00	0.00	0.00	0.00	0.02
**S.Rhone**	0.00	0.00	0.00	0.00	0.00	0.00	0.00	0.00	0.00	0.00	0.00	0.02
**S.Rough:i,v:1,2**	0.00	0.00	0.00	0.00	0.00	0.00	0.00	0.00	0.00	0.00	0.00	0.02
**S.Yovokome**	0.00	0.00	0.00	0.00	0.00	0.00	0.00	0.00	0.00	0.00	0.00	0.02
